# Supplementary material for: HIVprotI: an integrated web based platform for prediction and design of HIV proteins inhibitors
Source: J Cheminform. 2018 Mar 9;10:12. doi: 10.1186/s13321-018-0266-y (PMC5845081; doi:10.1186/s13321-018-0266-y)
Supplement: Supplementary file 2 — Additional file 2. Source code of HIVProtI web server. [file 13321_2018_266_MOESM2_ESM.zip › HIVprotI_Source-code/hivprotI/weka-3-6-6/doc/weka/attributeSelection/LFSMethods.Link2.html]

LFSMethods.Link2


---


|  |  |  |
| --- | --- | --- |
| **Overview** \| **Package** \| **Class** \| **Tree** \| **Deprecated** \| **Index** \| **Help** \| **Weka's home** | |  |
| **PREV CLASS**   **NEXT CLASS** | **FRAMES**    **NO FRAMES**     **All Classes** |  |
| SUMMARY: NESTED \| FIELD \| CONSTR \| METHOD | DETAIL: FIELD \| CONSTR \| METHOD |  |


---


## weka.attributeSelection Class LFSMethods.Link2

```
java.lang.Object
  weka.attributeSelection.LFSMethods.Link2
```

**All Implemented Interfaces:**: java.io.Serializable, RevisionHandler

**Enclosing class:**: LFSMethods

---

``` public class LFSMethods.Link2 extends java.lang.Object implements java.io.Serializable, RevisionHandler ```

Class for a node in a linked list. Used in best first search.
Copied from BestFirstSearch

**Author:**
:   Mark Hall (mhall@cs.waikato.ac.nz)

**See Also:**: Serialized Form

---

**Constructor Summary**

`LFSMethods.Link2(java.lang.Object[] data,
double mer)`


| **Method Summary** | |
| --- | --- |
| `java.lang.Object[]` | `getData()`             Get a group |
| `java.lang.String` | `getRevision()`             Returns the revision string. |
| `java.lang.String` | `toString()` |

**Methods inherited from class java.lang.Object**

`equals, getClass, hashCode, notify, notifyAll, wait, wait, wait`

**Constructor Detail**

### LFSMethods.Link2

```
public LFSMethods.Link2(java.lang.Object[] data,
                        double mer)
```


**Method Detail**

### getData

```
public java.lang.Object[] getData()
```

:   Get a group

---


### toString

```
public java.lang.String toString()
```

:   **Overrides:**: `toString` in class `java.lang.Object`

---


### getRevision

```
public java.lang.String getRevision()
```

:   Returns the revision string.

    :   **Specified by:**: `getRevision` in interface `RevisionHandler`
    :   **Returns:**: the revision


---


|  |  |  |
| --- | --- | --- |
| **Overview** \| **Package** \| **Class** \| **Tree** \| **Deprecated** \| **Index** \| **Help** \| **Weka's home** | |  |
| **PREV CLASS**   **NEXT CLASS** | **FRAMES**    **NO FRAMES**     **All Classes** |  |
| SUMMARY: NESTED \| FIELD \| CONSTR \| METHOD | DETAIL: FIELD \| CONSTR \| METHOD |  |


---
